# 

**Published:** 1902-01

**Authors:** 


LEADS VETERINARY JOURNALISM IN AMERICA.

Vol. XXIII. JANUARY, 1902. No. 1

PUBLISHED MONTHLY AT $3 A YEAR. SINGLE COPIES, 30 CENTS.

FOREIGN SUBSCRIPTIONS $3.50 PER YEAR.

### THE JOURNAL OF COMPARATIVE MEDICINE AND VETERINARY ARCHIVES

EDITED AND PUBLISHED BY
W. HORACE HOSKINS, D.V.S.,

ALL COMMUNICATIONS AND PAYMENTS SHOULD BE ADDRESSED TO
**Dr. W. Horace Hoskins, Managing Editor, 3452 Ludlow St., Philadelphia, Pa.**
Copies may be had on order of all news-dealers at home and abroad.

AN ADVERTISING MEDIUM UNEQUALLED IN THE VETERINARY FIELD

